# Characteristics of intellectual development in children diagnosed with arthrogryposis multiplex congenita

**DOI:** 10.3389/fpsyg.2026.1704823

**Published:** 2026-07-20

**Authors:** Maria Koriakina, Anastasia Urentcova, Yulia Burdukova, Olga Agranovich, Evgeny Blagovechtchenski

**Affiliations:** 1 Affective Psychophysiology Laboratory, Institute of Health Psychology, HSE University, Saint Petersburg, Russia; 2 H.Turner National Medical Research Center for Children’s Orthopedics and Trauma Surgery of the Ministry of Health of the Russian Federation, St. Petersburg, Russia

**Keywords:** arthrogryposis multiplex congenita, child development, cognitive skills, intelligence, motor disorders, motor skills

## Abstract

This study investigates how congenital motor disorders affect cognition by analyzing intelligence in children with arthrogryposis multiplex congenita (AMC), a condition featuring joint contractures without CNS involvement. Unlike other motor disorders, AMC offers a unique model to study movement restriction’s isolated cognitive effects. The study included 32 children diagnosed with AMC and a control group of 33 healthy children. To assess cognitive development, we administered the Culture-Free Intelligence Test (CFIT; GFT 2) to measure fluid intelligence, along with the Block Design, Similarities, and Vocabulary subtests from the Wechsler Intelligence Scale for Children (WISC) to evaluate visual–spatial, abstract-logical, and verbal intelligence, respectively. Comparative assessments of verbal and non-verbal intelligence in 6–15-year-olds revealed lower general and verbal-logical scores in AMC children, with gaps diminishing by adolescence. Non-verbal disparities emerged later, suggesting compensatory reorganization. Key finding: hand motor impairment—not overall severity—best predicted cognitive delays, emphasizing object manipulation’s role in early development. Results advocate for dual motor-cognitive interventions and refined diagnostic frameworks to distinguish AMC’s neuropsychological profile from other locomotor disorders.

## 1 Introduction

The study of the relationship between motor and cognitive development remains a relevant topic. Existing evidence suggests that active manipulation of objects plays a crucial role in the child’s adaptation to the environment (Bjorklund, 2022) and serves as a turning point in the transition from concrete actions to abstract thinking (Gilead et al., 2020). Importantly, the presence of motor impairments may be the cause of impaired cognitive development (Tolmacheva et al., 2024; Bhat, 2021; Blagoveschenskiy et al., 2018; Ntoumanis et al., 2021).

The present study analyzes intelligence characteristics in primary and middle school children diagnosed with Arthrogryposis Multiplex Congenita (AMC), a non-progressive condition affecting motor neurons in the spinal cord, characterized by muscle atrophy/hypertrophy and joint contractures (Niles et al., 2019; Rozhdestvenskiy et al., 2012). While it’s exact etiology remains unclear, AMC likely stems from fetal akinesia due to spinal motor neuron abnormalities.

Unlike progressive neuromuscular diseases, AMC manifestations remain stable postnatally. In contrast to cerebral palsy (CP), AMC does not involve pyramidal/extrapyramidal tracts, excluding spasticity or dystonia (Niles et al., 2019). This unique profile makes AMC an ideal model for studying cognitive development without confounding CNS damage.

AMC presentation varies significantly in severity. Most children require assistive devices or surgery for mobility, and while some achieve full motor recovery post-rehabilitation, others retain residual impairments (Hamdy et al., 2019). Crucially, upper limb involvement often prevents the independent execution of daily activities, such as eating or writing, forcing compensatory body use (Agranovich et al., 2021; Steen et al., 2018). Thus, children with AMC typically represent a heterogeneous group in terms of motor impairment, as the condition manifests in varying degrees of severity. A key characteristic of AMC is the presence of flexion-extension contractures in the joints of the upper and lower limbs, which can occur in different combinations (Cachecho et al., 2024).

Existing research on AMC cognitive profiles reveals working memory deficits (Bartonek et al., 2021), attention and memory impairments (Koriakina et al., 2021), and challenges in executive function, particularly in verbal fluency and semantic associations (Koriakina et al., 2025). The results showed that the cognitive functions of children with motor impairment were reduced, primarily in auditory and visual memory, as well as auditory attention. Lower scores were also found for visual and figurative thinking in children with motor disorders. A comparison of memory development indicators reveals the most significant disparities in children with motor disorders aged 8 to 10 (primary school age) (Koriakina et al., 2021). However, it should be noted that these findings were derived from mixed samples where children with AMC were analyzed alongside those with obstetric brachial plexus palsy (OBPP), potentially obscuring condition-specific cognitive profiles. This methodological approach may have limited the ability to identify AMC-specific developmental patterns, as the two groups differ fundamentally in etiology (congenital vs. acquired injury).

At the same time, in the context of developing verbal-logical thinking and intelligence, as measured by J. Raven’s “Progressive Matrix Scale,” children with upper limb motor limitations did not show a statistically significant difference when compared to their typically developing peers (Koriakina et al., 2021). In this regard, a recent study by Korneev, A. A., is crucial because it highlights that, based on contemporary theories of intelligence, the three components of this test pertain to different functions and aspects of intelligence (Korneev et al., 2024), which may have impacted the results. Given these novel findings, the present study will examine various forms of intelligence, including verbal intelligence. This is because children with AMC often exhibit delayed speech development, making it crucial to pay attention to this aspect (Agranovich et al., 2021; Koriakina et al., 2021).

During the comparative analysis of children with AMC and those with OBPP, it was concluded that, despite a significant difference in motor development between representatives of similar diseases, the indicators of cognitive function development did not show a significant difference. At the same time, both groups showed significantly lower indicators of memory and cognitive development compared to their healthy peers. As a result, it was suggested that the presence of musculoskeletal disorders may play a more significant role than the manifestation of motor disorders (Blagovechtchenski et al., 2023), which correlates with another conclusion regarding the cognitive development of children with musculoskeletal disorders (Clearfield, 2004).

Some data show that children with AMC need a personalized interdisciplinary approach in habilitation (Nematollahi et al., 2025), and physiotherapy alone is insufficient as a standalone intervention (Lobo et al., 2013). Further research in this area is therefore necessary, particularly concerning the cognitive development of children with AMC. Another issue is that other studies include this group of children in a broader category of children with locomotor disorders. However, children with AMC exhibit distinct motor disorder characteristics and do not have concomitant central nervous system lesions.

This article examines the notion that congenital motor impairments in children with AMC, particularly limitations in hand function, impact their cognitive development, specifically verbal and nonverbal intelligence, across various age groups. By examining these cognitive domains in this population, the study aims to elucidate the specific impact of motor restrictions on cognition, to describe age-related patterns in cognitive functioning, and to emphasize the importance of AMC-specific research in informing targeted habilitation strategies. While a cross-sectional design does not allow for definitive conclusions about developmental trajectories, identifying differences between younger and older participants may offer preliminary insights that can guide future longitudinal investigations into potential compensatory processes. Based on our own and literary data, we can assume that severe congenital motor disorders can also affect the intellectual development of a child.

**Figure 1:**
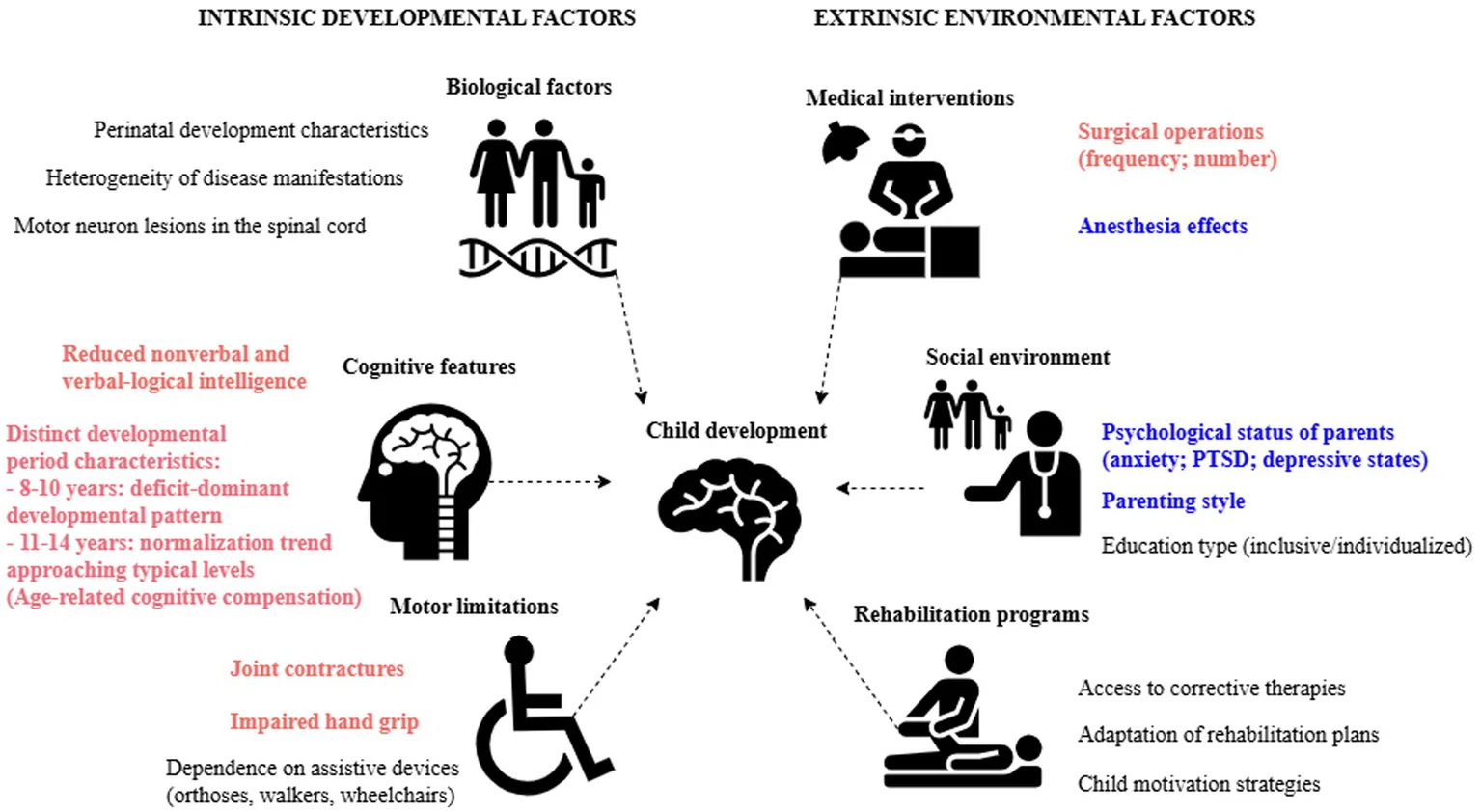
Factors influencing the development of children with AMC. Figure 1 presents a schematic classification of factors influencing the development of children with motor disorders (arthrogryposis and paresis), along with key observed patterns of their interaction. The factors are categorized into two interrelated groups: intrinsic (endogenous) and extrinsic (exogenous). Critically, factors highlighted in red represent novel investigative foci: the under recognized neurocognitive sequelae of repeated surgical stress and the cascading effects of early motor deprivation on higher-order cognition. The analysis of evidence for critical period plasticity in AMC-specific rehabilitation. These domains were directly addressed in the present study. Blue-highlighted elements identify emerging priority domains requiring systematic integration into clinical paradigms, particularly the longitudinal effects of anesthesia exposure timing and stress associated with surgery, as well as the psychological state of parents. These domains were not directly addressed in the present study.

The presented study highlights the complex, multidirectional interplay between intrinsic and extrinsic factors in shaping cognitive and motor development in children with AMC (Agranovich et al., 2021; Koriakina et al., 2021; Bartonek et al., 2021; Koriakina et al., 2025) (see Figure 1). In Figure 1, the key findings of this study—the interplay between intrinsic and extrinsic factors in AMC—are highlighted in red. It is important to note that while this figure illustrates the wide range of factors potentially influencing development in children with AMC (Niles et al., 2019; Cachecho et al., 2024; Nematollahi et al., 2025), the present study focuses specifically on the assessment of cognitive abilities. A comprehensive evaluation of all contributing factors—including detailed family, social, and therapeutic variables—was beyond the scope of this study due to practical constraints inherent to clinical data collection (Steen et al., 2018; Hamdy et al., 2019). Future research should aim to incorporate these domains more systematically (Lobo et al., 2013; Farran et al., 2021).

## 2 Methods

### 2.1 Participants

The experimental group comprised 32 patients diagnosed with AMC who were of primary and secondary school age and did not have accompanying central nervous system lesions. The experimental group included 32 children with arthrogryposis (20 with the generalized and 12 with the distal form), divided into younger (8–10 years) and older (11–14 years) age subgroups. Among these, 12 were aged 8 to 10 years, and 20 were aged 11 to 14 years. Most participants (75%) had moderate disease severity, with all children demonstrating multiple joint contractures of the upper and lower extremities, including the hands. The number of surgeries per patient ranged from 3 to 16, with a trend toward earlier surgical interventions in the younger group.

The functional abilities of the children varied significantly: 59% retained the ability to walk independently, while 41% required the use of assistive devices. Hand grip was preserved in 56% of participants. Notably, only 22% of children received regular corrective assistance, which was significantly lower in the older group (15% vs. 33% in the younger group). (see Supplementary Tables S1–S5 for details).

All children diagnosed with AMC had undergone surgery to restore motor activity (see Supplementary Table S11). The analysis revealed distinct surgical patterns within the cohort. Older patients underwent an average of 11 surgeries; most (72%) required 6 or more surgeries, with a maximum of 15 (in one case). The number of surgeries ranged from 2 to 16. Four patients had between 2 and 6 surgeries, five had between 7 and 11, and three had between 12 and 16. Therefore, the majority (8 out of 12, or 67%) underwent 7 or more surgeries, which explains the reported average of 6–7. Despite the surgical intervention and rehabilitation measures, none of the patients achieved the level of motor function typical for healthy children. This allowed all participants to be grouped for analysis, as their postoperative functional abilities were comparable to one another, but remained lower than the age norm.

The comparative analysis group consisted of 33 children from general education institutions, matched for sex and age, who did not have musculoskeletal or central nervous system disorders, nor any other additional medical conditions. This group also contained 12 children in the primary school age range and 21 in the secondary school age bracket.

The study’s methodological toolkit included various diagnostic techniques designed to assess the levels of development of fluid intelligence, verbal and non-verbal intelligence components, spatial intelligence, and verbal-logical thinking.

Severity of motor disability was classified as mild, moderate, or severe based on a multidisciplinary clinical assessment performed by orthopedic specialists. This assessment integrated the following parameters: the number and severity of joint contractures, the range of active and passive motion, the level of functional independence in daily activities (e.g., self-care, mobility), and data from medical imaging (Agranovich et al., 2021). The final classification reflected a consensus among the treating physicians and was documented in the patients’ medical records.

All participants were recruited from the inpatient departments. Children in the AMC group were invited to participate during their routine orthopedic follow-up visits or rehabilitation admissions. The control group consisted of typically developing children recruited from local schools and community settings in the same geographical region. The experimental and the control groups do not have statistically significant differences in age.

Prior to participation, parents or legal supervisors of all children received detailed information about the study aims and procedures and provided written informed consent. Additionally, verbal assent was obtained from each child before testing began. Children were assured that they could stop participating in the experiment at any time without any consequences for their medical care or school activities.

Assessments were conducted individually in a quiet room by trained psychologists with experience in pediatric assessment. The duration of each session was approximately 60–90 min, with breaks provided as needed to prevent fatigue. The four subtests (CFIT, Block Design, Similarities, and Vocabulary) were performed in a fixed order: Vocabulary and Similarities (verbal tasks) were alternated with Block Design and CFIT (nonverbal tasks) to maintain participant engagement and minimize fatigue. No counterbalancing was applied, as the tasks were relatively brief and the fixed order was considered appropriate for this clinical sample.

A full performing of all WISC subtests was considered too demanding for many children in the clinical group due to fatigue associated with their motor condition and concurrent medical procedures. Therefore, a focused set of three subtests was selected to assess key cognitive domains while minimizing participant burden. The subtests were chosen based on their high factor loadings on the Verbal Comprehension Index (Vocabulary and Similarities) and Perceptual Reasoning Index (Block Design).

### 2.2 A culture-free intelligence test Cattell, R. B

In R. Cattell developed the Culture-Free Intelligence Test (CFIT) to minimize the influence of cultural, educational, and linguistic factors on the assessment of cognitive abilities. The technique is based on a nonverbal task format that analyzes visual patterns, spatial relationships, and logical sequences, allowing it to be applied across various ethnocultural groups with minimal modifications (Cattell, 1940).

This test measures fluid intelligence—the innate ability for abstract logical thinking, pattern recognition, and solving novel problems independently of acquired knowledge. Fluid intelligence is characterized by an innate ability for abstract logical thinking, pattern identification, solving new problems without relying on previous experience, and adaptive learning in unfamiliar conditions.

Cattell associated this intelligence component with biological factors (CNS functioning, neuroplasticity) and considered it relatively independent of education and culture.

To measure children’s general fluid intelligence level using a variant of the test GFT 2. Non-verbal intelligence describes thinking skills and problem-solving abilities that do not fundamentally require verbal language production and comprehension. This type of intelligence involves manipulating or problem-solving with visual information. It may vary in the internalized, abstract, or conceptual reasoning and motor skills required to complete a task (Anagnostou et al., 2013).

The first subtest, “Addition,” encompasses twelve tasks. Each task presents three figures on the left side arranged in a specific logical sequence. Participants must analyze the logic governing the changes within this sequence and select the figure that can logically extend the identified pattern from a set of five options.

The second subtest, titled “Classification,” comprises fourteen tasks. Each task presents five figures, four of which share a common characteristic, while the fifth is different. The criteria linking these figures may include attributes such as shape, quantity, color, or their spatial relationships.

The third subtest, “Matrix,” comprises 12 tasks that require logical image comparison. A key feature of this subtest is the need to thoroughly analyze the attributes simultaneously from horizontal, vertical, and diagonal perspectives.

The fourth subtest,” Topology,” consists of eight tasks. Each series includes a reference figure depicting simple geometric shapes and a point. To determine the solution, it is essential to analyze the point’s positioning in relation to all figures shown in the reference image. Subsequently, it is necessary to identify one that facilitates the analogous placement of the fact among the remaining five figures, as observed in the reference figure. The shared characteristics among the figures may consist of shape, quantity, color, spatial relationships, and various combinations thereof.

### 2.3 Wechsler intelligence scale for children. Kohs block design subtest

This subtest assesses visual–spatial intelligence and visuo-constructive skills—the ability to analyze and synthesize visual information and to reproduce spatial relationships. This test measures the extent to which spatial analysis, synthesis, and motor coordination are developed; it is the most informative tool for assessing subjects’ level of non-verbal intelligence. The subject is presented with pictures and cubes with patterns; the task is to match the cubes to the picture (Wechsler, 1949).

### 2.4 Wechsler intelligence scale for children. Similarities subtest

This subtest measures abstract verbal reasoning and logical thinking—the capacity to identify essential relationships between concepts. The test aims to reveal the ability of abstract-logical thinking. This type of thinking involves identifying an object’s essential properties and connections, and abstracting from other, less important ones. The indicators obtained from performing this test can also indirectly reflect the level of general knowledge and ideas about the world around us, as successful performance of the test tasks requires the subject to possess a specific educational background. The child is presented with a couple of words, and the task is to name the common feature correctly (Wechsler, 1949).

### 2.5 Wechsler intelligence scale for children. Vocabulary subtest

This subtest assesses verbal intelligence and lexical knowledge—the breadth of vocabulary and the ability to articulate word meanings. It also indirectly reflects the child’s general knowledge and educational background. High verbal–linguistic intelligence is associated with lexical knowledge, verbal memory, the ability to understand and manipulate syntax, and the capacity to utilize symbolic and abstract language (Augustyniak, 2011). Additionally, this subtest enables us to assess the test taker’s vocabulary, speech culture, and cognitive skills. It is the least dependent on external factors influencing the test (such as injuries and illnesses) and can characterize the respondent’s premorbid intelligence. The subtest presents the examinee with a series of increasingly complex words (e.g., from simple nouns like “apple” to abstract concepts like “justice”), which they must define orally in their own words. The examiner records verbatim responses, which are then scored against standardized criteria that evaluate definitional accuracy, conceptual depth (the ability to identify essential features rather than superficial attributes), and linguistic precision. Higher scores reflect greater lexical knowledge and verbal reasoning ability (Wechsler, 1949).

For all WISC subtests, scaled scores (M = 10, SD = 3) were used in the statistical analyses. These scores are age-corrected and allow for direct comparison across different age groups. For the CFIT (Culture-Free Intelligence Test), standard scores (IQ points: M = 100, SD = 15) were calculated according to the manual guidelines (Wechsler, 1949).

## 3 Statistical analysis

Statistical analyses were performed using IBM SPSS Statistics (version 21). To account for multiple comparisons, the Bonferroni correction was applied. Since the distribution of test results across various parameters violated the normality assumption (as determined by the Kolmogorov–Smirnov test, *p* < 0.05), the non-parametric Mann–Whitney U test was utilized to compare independent samples (see Supplementary Table S6).

Prior to data collection, a power analysis was conducted using GPower software (Faul et al., 2007). Based on an anticipated medium effect size (Cohen’s d = 0.5), *α* = 0.05, and power = 0.80, the required sample size was estimated at approximately 64 participants per group for t-tests. Due to the rarity of AMC, achieving this sample size was not feasible; therefore, the study should be considered exploratory, and results should be interpreted with caution. Post-hoc power calculations are reported in the Results section where applicable.

In addition to *p*-values, effect sizes (Cohen’s d for t-tests and partial η^2^ for ANOVA) are reported to facilitate interpretation of the magnitude of group differences. According to conventional benchmarks (Cohen, 1988), *d* = 0.2 is considered a small effect, *d* = 0.5 a medium effect, and *d* = 0.8 a large effect.

## 4 Results

### 4.1 Results comparing a group of children with AMC with a control group of normotypic children

#### 4.1.1 Results of the culture-free intelligence test Cattell, R. B

**Figure 2:**
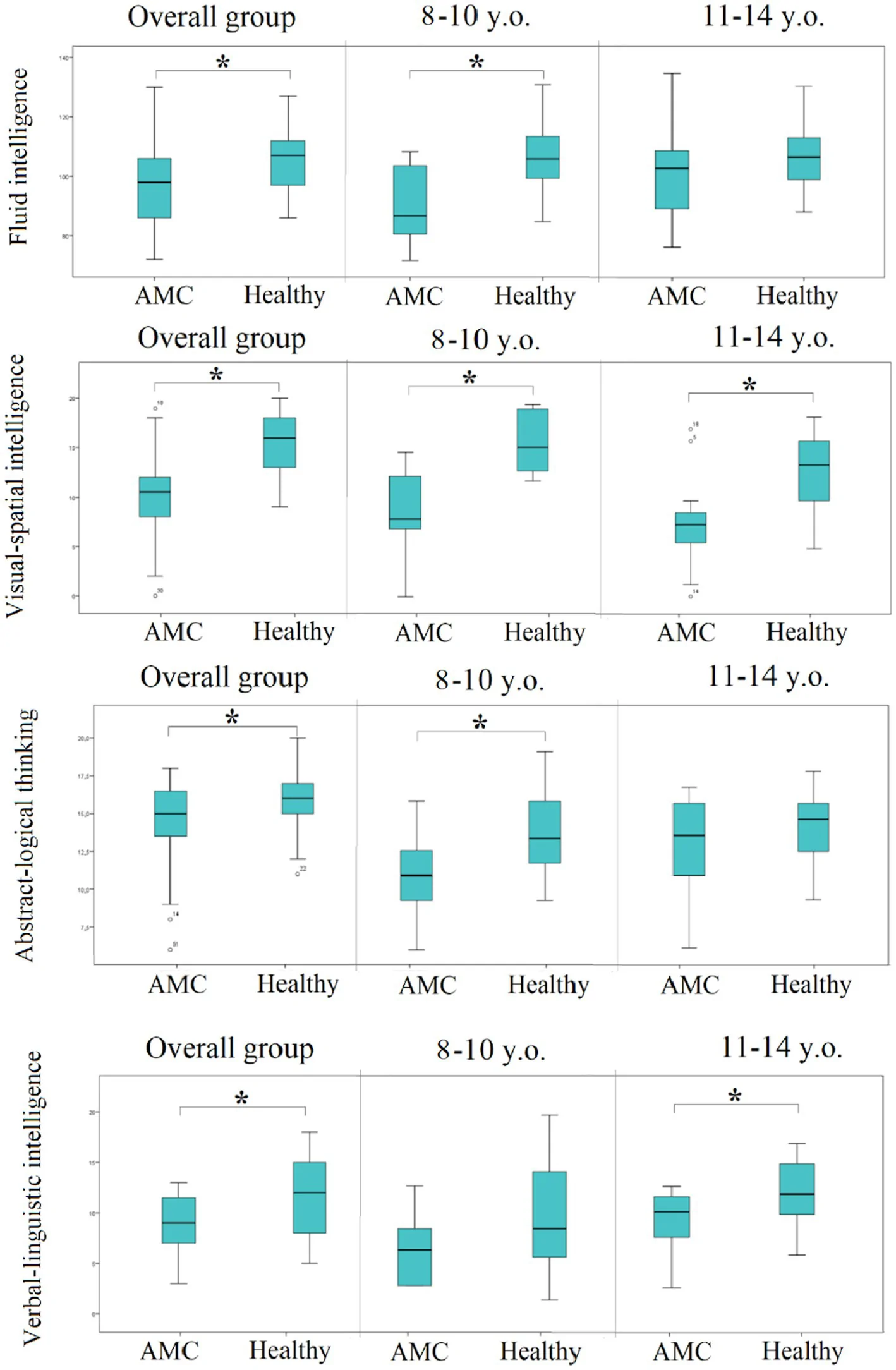
Results comparing a group of children with AMC with a control group of normotypic children (see Supplementary Tables S6, S7).

Analysis of the results of Cattell’s test revealed statistically significant differences in children with AMC compared to the healthy children (Z = 3.12, *p* = 0.003) (see Figure 2 and Supplementary Table S8). In the group of children with AMC, the mean score was 96.72 (SD = 14.34), which was significantly lower than that of the control group of healthy children, where the mean score was 106.82 (SD = 12.03).

When the data were divided by age group, it was found that children with AMC (*μ* = 91.33, SD = 14.099) performed significantly lower than healthy peers (*μ* = 106.82, SD = 13.49) in the younger group (*Z* = 3.05, *p* = 0.004).

At the same time, in the middle school age group, the level of fluid intelligence of children with arthrogryposis was 99.95 (SD = 13.83), while that of the control group was 105.24 (SD = 11.14). However, the significance level in this case was *Z* = 1.35, *p* = 0.184, indicating no statistically significant difference between the groups. At the same time, statistically significant differences in fluid intelligence were also observed between children of different ages with AMC, with children aged 11–14 demonstrating higher indicators. A statistically significant negative correlation was found between the number of surgical procedures and fluid intelligence scores (*Z* = −2.01, *p* = 0.045).

#### 4.1.2 Results of the Wechsler intelligence scale for children. Kohs block design subtest

The Koch’s Blocks subtest results showed significant differences between children with AMC and those in the control group (*Z* = 6.45, *p* < 0.001) (see Figure 2 and Supplementary Table S9). The analysis showed that the mean score of children with arthrogryposis was 10.09 (SD = 3.98), whereas it reached 15.61 (SD = 3.03) in typically developing children. This suggests a significantly lower visual intelligence and spatial analysis ability level in children with motor disorders than their peers.

When comparing age groups, differences persisted in the younger (*Z* = 5.82, *p* < 0.001) and middle-aged groups (*Z* = 4.10, *p* < 0.001): children with AMC had mean scores of 8.58 and 11.00, respectively, while the control groups had higher scores of 16.08 and 15.33.

Statistical analysis of the data has shown that children with AMC have an average level of non-verbal intelligence, and the ability to spatially analyze and synthesize, which is one and a half to two times lower than that of children without pathology, both in the sample as a whole and when comparing the two categories of children separately by age groups.

#### 4.1.3 Results of the Wechsler intelligence scale for children. Similarities subtest

The analysis results showed that children with AMC demonstrated reduced generalization and verbal-logical reasoning abilities compared to their typically developing peers (*Z* = 2.55, *p* = 0.013) (see Figure 2 and Supplementary Table S10). Overall, the mean score of the group with AMC was 14.38 (SD = 2.89). In contrast, the control group had a mean score of 16.00 (SD = 2.22).

Dividing the results by age group, the differences remain significant (*Z* = 2.18, *p* = 0.039) at the younger school age: children with AMC obtained an average of 14.42 (SD = 2.48), whereas children in the control group showed an average of 16.75 (SD = 2.61). However, the differences in the older age group were not statistically significant (*Z* = 2.31, *p* = 0.136): 14.35 in children with AMC and 15.57 in healthy children. No statistically significant differences were found between the groups of children with AMC in the younger and middle school age groups (*Z* = 2.51, *p* = 0.951).

Similar to the results of the fluid intelligence, an analysis of the data from the subtest focused on assessing verbal-logical thinking and the ability to identify standard features among different concepts confirmed statistically significant deficits in the younger age group compared to typically developing peers. Meanwhile, the middle school age group showed no statistically different results.

#### 4.1.4 Results of the Wechsler intelligence scale for children. Vocabulary subtest

The analysis revealed that children with AMC exhibit lower vocabulary and definition skills compared to their typically developing peers (*Z* = 3.03, *p* = 0.003) (see Figure 2 and Supplementary Table S11). The average score for the group with arthrogryposis was 9.19 (SD = 2.73), then, as in the control group, it was 11.70 (SD = 3.74).

Upon further analysis of the results by age category, the average score for children with AMC in the primary school age category was 8.58 (SD = 2.43), which is significantly lower than that of their normotypic peers, whose average score was 10.92 (SD = 4.21). Despite the overall lag, the gap was not significant in this age group (*Z* = 1.65, *p* = 0.110).

The situation changed in the group of secondary school-aged children: the average score of children with AMC was 9.55 (SD = 2.89), while that of their peers was 12.14 (SD = 3.47). Here, the difference turned out to be statistically significant (*Z* = 2.60, *p* = 0.013). These results indicate while scaled scores for verbal subtests remained relatively stable across age groups in children with AMC (indicating age-appropriate raw score growth), comparison with the control group revealed a widening gap in later childhood, suggesting that the rate of verbal development may not fully keep pace with typically developing peers.

The level of verbal intelligence in the nosological group is reduced; however, contrary to non-verbal intelligence, the more significant differences in average indicators were not found in the younger age group but in the older age group. At the same time, the maximum score for this substandard in either age category among children with AMC did not rise above 13. In contrast, the scale score could reach 17 or 18 points among neurotypically developing peers.

Meanwhile, the gap in mean scores for the tests assessing verbal intelligence (Similarities and Vocabulary subtests) turned out to be less than that for the Kohs Block design subtest, which estimates the non-verbal component of intelligence.

The largest effect sizes were observed for the Block Design subtest across all age groups (overall: *d* = −1.55; 8–10 years: *d* = −1.86; 11–14 years: *d* = −1.38), indicating very large differences between children with AMC and healthy controls in visuospatial processing. Medium to large effects were found for fluid intelligence (CFIT overall: *d* = −0.76), with the effect being particularly pronounced in the younger age group (*d* = −1.12). Vocabulary showed consistent medium to large effects across age groups (overall: *d* = −0.77), while Similarities demonstrated medium effects (overall: *d* = −0.63), with larger effects in younger children (d = −0.88).

### 4.2 The analysis of factors potentially influencing cognitive performance revealed selective associations

Disability severity level, classified as mild, moderate, or severe, showed no statistically significant effect on any cognitive outcome measure. It might be due to the small number of respondents observed and the uneven distribution of different disability level within the cohort. Therefore, this factor might demand further investigation on a larger sample of children. In contrast, the number of surgeries a child had undergone demonstrated age-dependent correlations. For the middle-school-age subgroup, a significant inverse correlation was found between the number of surgeries and scores on the Cattell IQ test, which measures fluid intelligence (Spearman’s rho = −0.453, *p* = 0.045). Conversely, for the elementary-school-age subgroup, a positive correlation was observed between the number of surgeries and performance on the “Vocabulary” subtest (rho = 0.656, *p* = 0.021). No significant correlations were found for the entire sample or for other cognitive domains. Furthermore, while the presence of contractures in the lower extremities or the loss of independent ambulation showed no significant link to cognitive scores, the preservation of hand grasp function was relevant. Specifically, within the elementary-school-age group, children with preserved hand grasp significantly outperformed those with impaired grasp on the “Kohs Block” test of spatial intelligence (Mann–Whitney U, *p* = 0.027). No other examined parameters, including gender, diagnosis type, or participation in regular corrective-developmental sessions, yielded significant results (See Supplementary Tables S12–S14).

## 5 Discussion

This study demonstrated a specific uniqueness in the development of cognitive abilities among children with AMC. On average, the level of non-verbal intelligence in the nosological group was lower than that of their healthy peers. However, this gap tends to narrow as the child matures. By middle school age, there are no statistically significant differences in fluid intelligence level between children with and without the pathology. These findings are consistent with studies demonstrating the hypothesis of neuroplasticity and compensatory mechanisms in early rehabilitation (Shi and Feng, 2022).

The results obtained allowed us to conclude that children with AMC have a lower rate of cognitive development and initially develop according to the deficient type. However, by the time they reach the secondary level of general education, the formation of the basic cognitive functions necessary for the learning process is complete (Ollikainen et al., 2024). Their intellectual level can often equal that of their healthy peers. This aligns with the study’s conclusion that there is a specificity to the development of spatial intelligence in children with physical disabilities, as well as a specificity to the errors they make on such tasks (Farran et al., 2021).

It is also important to note that this does not directly contradict the results of previous studies, which showed that children with AMC do not differ from their peers in terms of intelligence (Koriakina et al., 2021), as the last study employed the “Raven Matrix” intelligence assessment technique. A recently published study showed that three parts of the matrices in this technique are related to different groups of functions to varying degrees: the first part of the technique is most closely associated with the state of visual information processing functions, the second—with the state of processing of visual–spatial information and control functions, and the third is more related to the state of control functions (Korneev et al., 2024). This heterogeneity of the methodology can affect the results. As we can see from this study, the results of tests on various aspects of intelligence vary (such as comparing groups on verbal and non-verbal intelligence tests).

Regarding the development of visual and spatial intelligence, the children studied exhibit indicators significantly lower than those of their healthy peers, resulting in a notable gap at both age stages. Across the entire group and within different age groups, children with AMC performed on average one and a half to two times worse than their peers without pathologies. Meanwhile, a positive correlation was found between success in the spatial thinking test and overall cognitive function, which was statistically significant for the entire cohort and the group of primary school-aged children (*p* < 0.05). This finding may reflect the compensating influence of specific cognitive functions on deficient aspects of intelligence (Bertelli et al., 2018).

Notably, age-related patterns varied across subtests. While younger children with AMC showed relatively preserved verbal intelligence, adolescents caught up to their peers in verbal-logical tasks. According to the test designed to assess verbal and logical thinking, adolescents did not differ from their peers in the control group. All three tests on various aspects of intelligence showed the presence of significant differences that manifest themselves precisely at middle school age, which may indicate that the age of 11–12 years becomes essential in the formation of cognitive abilities and the general level of intelligence of children with AMC since it is at this time that various manifestations of the cognitive sphere acquire an integrated and formalized character (Hoorani et al., 2022).

This phase may reflect the integration of higher-order cognitive functions, influenced by motor-sensory deprivation, psychosocial factors, and educational transitions. Limited physical exploration due to AMC’s motor constraints may delay spatial and executive function development, as observed in children with cerebral palsy (Zielinski et al., 2014). Concurrently, increased social isolation or stigma during adolescence can exacerbate cognitive load, further impacting executive functioning (Prizeman et al., 2023). Finally, the shift toward abstract thinking in middle school aligns with heightened demands on working memory—a domain where children with motor impairments may struggle without targeted accommodations (RIOU et al., 2009).

Thus, this study confirms the developmental features of general verbal intelligence and individual cognitive functions of children with AMC. The statistical data analysis regarding the impact of various disease manifestation parameters on cognitive development in children with the studied pathology indicates the necessity for continued research in this area. The type of diagnosis and the severity of the restrictive manifestations of the disease did not show any connection with cognitive abilities. The influence of factors such as the availability of regular correctional and developmental classes on the annual schedule was not confirmed at any comparison level. However, the small number of children who received systematic correctional assistance could limit the possibilities of statistical analysis. At the same time, clinical and pedagogical experience reveals notable differences in the development of learning skills in children attending specialized educational institutions with comprehensive remedial support, including speech therapy, defectology, and psychological support, compared to children without such assistance. These findings highlight the necessity of longitudinal studies with larger samples to assess the long-term effects of rehabilitative interventions (Korobeynikov and Solovyova, 2020).

This study also examined the potential relationship between the studied cognitive parameters and disease factors in children with AMC. Various parameters are investigated, including the severity of the disease, the number of surgical interventions, the presence of contractures, the ability to walk independently, and participation in corrective and developmental activities. Statistical analysis revealed no significant differences in all major parameters, indicating that the type of diagnosis and severity of the diseases do not affect the level of intelligence and thinking in this group of children. This result confirms previously obtained data that the severity of motor impairments does not affect cognitive abilities as much as the presence of impairments (Blagovechtchenski et al., 2023).

Despite the absence of significant differences, correlations were observed, particularly concerning the number of operations performed. Specifically, a negative correlation was observed between the number of operations and nonverbal intelligence in middle school-age children. However, the result did not reach statistical significance for the whole sample. Contractures in lower limbs and the ability to walk independently were not statistically significant associations with test results. At the same time, significant correlations were found between the safety or restriction of hand grip and the results of tests of visual–spatial intelligence and verbal functions, confirming that children with motor impairments have lower indicators of visual–spatial intelligence and verbal intelligence, which also aligns with previously published data (Koriakina et al., 2024).

Since children with the pathology under study lag behind their healthy peers as they reach secondary school age, it can be assumed that the cognitive development of children with AMC is positively influenced simply by the act of schooling, irrespective of the form of education—whether individually, at home, in the classroom, or through a combination of these options (Saracho and Spodek, 2013).

We found that the presence of contractures in the lower limbs or the loss of independent walking did not reveal any correlations. Several specific correlations, confirmed by statistical criteria, were established regarding factors such as the number of operations performed by the child at the time of the examination and the preservation of hand grasping function. More significant is the data on the second factor, which demonstrates that the limitation of hand motor skills leads to lower results in visual–spatial intelligence, verbal-logical thinking, and verbal intelligence tasks. This finding is also consistent with previous data (Björelius and Tükel, 2017), which showed that in children with motor diseases affecting one hand, the results of semantic association tests were better than in children with both hands affected (Koriakina et al., 2024). Our results align with embodied cognition theory, suggesting that restricted motor experience in AMC may fundamentally shape cognitive development (Adolph and Hoch, 2019).

It is important to note that, despite the statistically significant differences between groups, the mean scaled scores of children with AMC on all WISC subtests were within the average range (scaled scores 8–12). This indicates that, as a group, children with AMC demonstrate age-appropriate cognitive abilities, even though their performance lags behind that of typically developing peers. This finding is clinically meaningful: while there may be a relative weakness compared to healthy children, absolute intellectual functioning remains within normal limits for the majority of the sample.

The present findings highlight the complex nature of developmental challenges in AMC, which extend beyond motor dysfunction to encompass cognitive factors. These results underscore the importance of comprehensive neuropsychological assessment in this population. Future research should investigate the interplay between motor, cognitive, and environmental factors longitudinally to better understand the mechanisms underlying these associations. Additionally, the potential role of specific factors (e.g., prolonged hospitalization, reduced social engagement, limited educational opportunities) needs further investigation in larger, more diverse samples. Such studies could help clarify whether the observed cognitive patterns reflect condition-specific characteristics or result from secondary environmental factors associated with the disorder.”

This evidence highlights the crucial need for personalized, multidisciplinary management strategies in this patient population (see Figure 1).

Three critical findings emerge from our results. First, the dissociation between motor impairment and cognitive potential is particularly noteworthy—while spinal motor neuron lesions and joint limitations constrain physical abilities, the preserved CNS integrity allows for near-normal baseline intellectual functioning, distinguishing this population from children with cerebral palsy. This suggests that targeted cognitive interventions could yield significant developmental benefits even in the presence of severe motor restrictions. Second, the identified critical periods (8–10 years for deficit manifestation vs. 11–14 years for compensation) underscore the neuroplastic potential during middle childhood, when intensive rehabilitation and educational support may be most effective. The negative correlation between multiple surgeries and nonverbal intelligence raises essential questions about the cognitive costs of aggressive medical interventions, warranting further investigation into optimal timing and alternative approaches. While the study does not establish causal relationships, we propose that the identified correlation might be explained by the cumulative effect of psychological stress. Each new preoperative waiting period may increase the child’s anxiety, and this accumulated stress could negatively affect cognitive functions, particularly during critical developmental periods.

Finally, the compensatory role of environmental factors—particularly inclusive education and adapted rehabilitation—emphasizes the importance of ecological approaches that address both physical constraints and psychosocial needs. These findings collectively suggest the need for integrated care models that synchronize medical management with cognitive and social support across various stages of development. Future research should explore longitudinal trajectories to better understand the cumulative effects of these interacting factors on functional outcomes in adulthood.

### 5.1 Limitations

A key limitation of the study concerns the sample size. Both the clinical group of children with AMC and the healthy control group were relatively small, which may limit the statistical power of the between-group comparisons and the generalizability of the findings. It is important to acknowledge that while we collected preliminary data on educational placement and non-medical interventions (e.g., occupational therapy, speech therapy, psychological support, parent training), these variables were not included in the main statistical analyses for two primary reasons. First, the sample was highly heterogeneous regarding the type, intensity, and duration of interventions, making it impossible to create meaningful comparison groups. Second, only three children in the sample attended specialized correctional classes, which was an insufficient number for subgroup analysis. Regarding educational format, approximately half of the children received individual education; however, this factor did not show significant correlations with cognitive outcomes in our preliminary exploratory analyses. Future studies with larger, more homogeneous samples are needed to systematically investigate the role of educational and therapeutic variables in cognitive development.

## 6 Conclusion

The level of general non-verbal intelligence and verbal-logical thinking in children with AMC at primary school age is lower than in children from the control group. However, by the time they progress to the general stage of basic education, the formation of cognitive functions necessary for learning is complete, and their intellectual level can be leveled out. Children with AMC have significantly lower indicators of visual and spatial intelligence than the control group, regardless of age. Regarding verbal intelligence, children with AMC do not differ from their peers during primary school and show significantly lower rates in middle school. This may indicate that compensation for particular cognitive functions with age may decrease other parameters. Statistically significant correlations have been established between intelligence development and indicators such as the number of surgical operations performed, which may demonstrate the effect of anesthesia and surgical interventions on a child’s intelligence. Statistically significant correlations have been established between intelligence development and indicators such as grasping hand function. This implies that the limitations of hand motor skills lead to lower results in tasks on visual and spatial intelligence, which the theory of embodied cognition can explain. The necessity for early cognitive interventions is especially pronounced in the context of developing visual–spatial skills. It is imperative to optimize re/habilitation process to mitigate its impact on cognitive function.

## Data Availability

The original contributions presented in the study are included in the article/Supplementary material, further inquiries can be directed to the corresponding author.
