# Supplementary material for: Characteristics of intellectual development in children diagnosed with arthrogryposis multiplex congenita
Source: Front Psychol. 2026 Jul 20;17:1704823. doi: 10.3389/fpsyg.2026.1704823 (PMC13429483; doi:10.3389/fpsyg.2026.1704823)
Supplement: Supplementary file 1 [file Supplementary_file_1.DOCX]

The sample included children with congenital multiple arthrogryposis:

Supplementary Table 1. By the severity of the disease:

| Age group | Disease severity | | |
| --- | --- | --- | --- |
|  | mild | medium | severe |
| 8-10 y.o. | 1 | 8 | 3 |
| 11-14 y.o. | 4 | 16 | - |
| Overall | 5 | 24 | 3 |

Supplementary Table 2. By the presence of contractures in the joints:

| Age group | Presence of contractures | | |
| --- | --- | --- | --- |
|  | In the lower limbs | In the upper limbs | In the wrists |
| 8-10 y.o. | 9 | 10 | 10 |
| 11-14 y.o. | 16 | 15 | 15 |
| Overall | 25 | 25 | 25 |

Supplementary Table 3. By the preservation of independent walking function:

| Age group | Independent walking | |
| --- | --- | --- |
|  | Preserved | Possible only with the use of special devices |
| 8-10 y.o. | 7 | 5 |
| 11-14 y.o. | 12 | 8 |
| Overall | 19 | 13 |

Supplementary Table 4. By the preservation of hand grip function;

| Age group | Hand grip | |
| --- | --- | --- |
|  | Preserved | Limited or impossible |
| 8-10 y.o. | 5 | 7 |
| 11-14 y.o. | 13 | 7 |
| Overall | 18 | 14 |

Supplementary Table 5. By the availability of remedial and developmental classes:

| Age group | Availability of remedial and developmental classes | |
| --- | --- | --- |
|  | + | - |
| 8-10 y.o. | 4 | 8 |
| 11-14 y.o. | 3 | 17 |
| Overall | 7 | 25 |

Supplementary Table 6. Statistical analysis tables


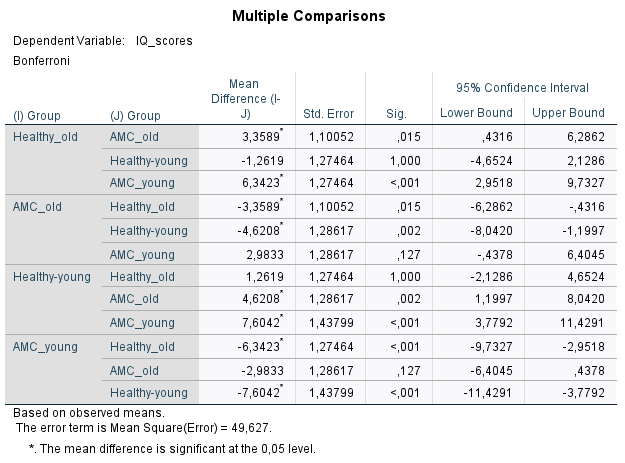


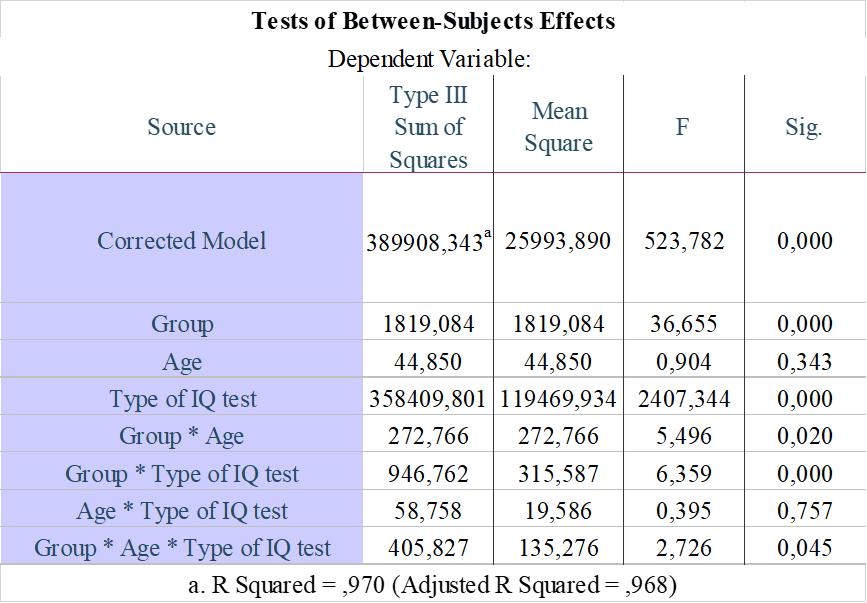


Supplementary Table 7. Three-Way ANOVA Results for Intelligence Test Performance: Main Effects and Interactions of Group (AMC vs Control), Age (Younger vs Middle School) and Test Type (Cattell, Kohs Blocks, Similarities, Vocabulary)


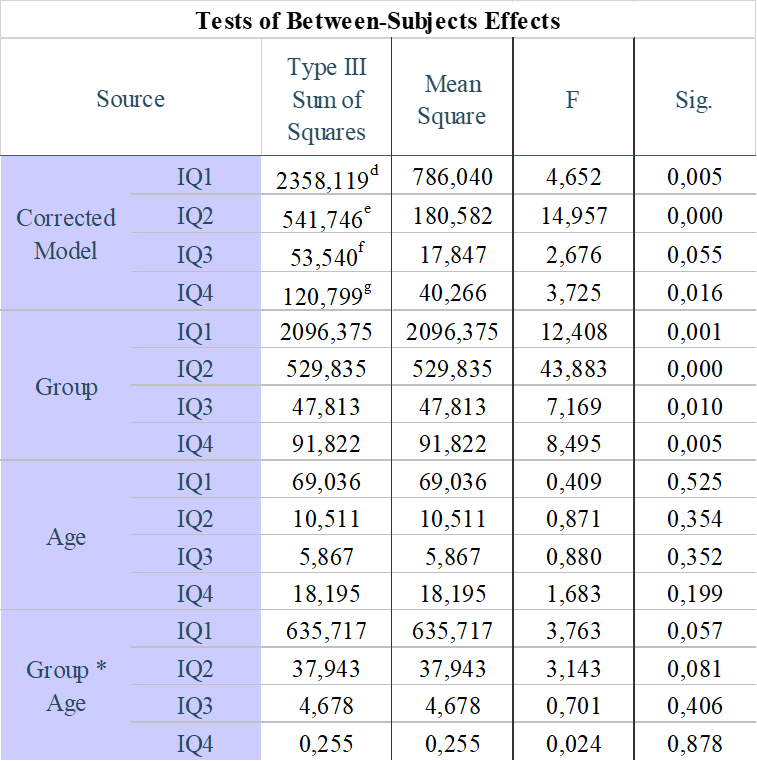


Supplementary Table 8**.** Between-Subjects Effects Analysis of Intelligence Tests:

Group Comparisons (AMC vs. Healthy) Across Age Strata and Full Sample.

| Type of IQ test | Group | | μ | SD | p-value |
| --- | --- | --- | --- | --- | --- |
| Culture-Free Intelligence Test | Overall | AMC | 96,72 | 14,34 | **р=0,003** |
|  |  | Healthy | 106,82 | 12,03 |  |
|  | 8-10 y.o. | AMC | 91,33 | 14,099 | **р=0,004** |
|  |  | Healthy | 106,82 | 13,49 |  |
|  | 11-14 y.o | AMC | 99,95 | 13,83 | р=0,184 |
|  |  | Healthy | 105,24 | 11,14 |  |

Supplementary Table 9**.** Summary data of the Wechsler Intelligence Scale for Children, Kohs Block Design subtest results when comparing the groups “AMC” and “Healthy” by two age groups and the whole sample.

| Type of IQ test | Group | | μ | SD | p-value |
| --- | --- | --- | --- | --- | --- |
| «Kohs Block Design subtest» | Overall | AMC | 10,09 | 3,98 | **р=0,000** |
|  |  | Healthy | 15,61 | 3,03 |  |
|  | 8-10 y.o. | AMC | 8,58 | 4,66 | **р=0,000** |
|  |  | Healthy | 16,08 | 3,23 |  |
|  | 11-14 y.o | AMC | 11,00 | 3,31 | **р=0,000** |
|  |  | Healthy | 15,33 | 2,96 |  |

Supplementary Table 10**.** Summary data of the Wechsler Intelligence Scale for Children, Similarities subtest results when comparing the groups “AMC” and “Healthy” by two age groups and the whole sample.

| Type of IQ test | Group | | μ | SD | p-value |
| --- | --- | --- | --- | --- | --- |
| «Similarities subtest» | Overall | AMC | 14,38 | 2,89 | **р=0,013** |
|  |  | Healthy | 16,00 | 2,22 |  |
|  | 8-10 y.o. | AMC | 14,42 | 3,09 | **р=0,039** |
|  |  | Healthy | 16,75 | 2,01 |  |
|  | 11-14 y.o | AMC | 14,35 | 2,85 | р=0,136 |
|  |  | Healthy | 15,57 | 2,27 |  |

Supplementary Table 11. Summary data of the Wechsler Intelligence Scale for Children, Vocabulary subtest results when comparing the groups “AMC” and “Healthy” by two age groups and the whole sample.

| Type of IQ test | Group | | μ | SD | p-value |
| --- | --- | --- | --- | --- | --- |
| «Vocabulary subtest» | Overall | AMC | 9,19 | 2,73 | **р=0,003** |
|  |  | Healthy | 11,70 | 3,74 |  |
|  | 8-10 y.o. | AMC | 8,58 | 2,43 | р=0,110 |
|  |  | Healthy | 10,92 | 4,21 |  |
|  | 11-14 y.o | AMC | 9,55 | 2,89 | **р=0,013** |
|  |  | Healthy | 12,14 | 3,47 |  |

Supplementary Table 12. The number of surgeries in the patient group.

| The number of surgeries | 8-10 y.o group | 11-14 y.o group | All patient |
| --- | --- | --- | --- |
| from 2 to 6 | 4 | 6 | 10 |
| from 7 to 11 | 5 | 12 | 17 |
| from 12 to 16 | 3 | 2 | 5 |

Supplementary Table 13. Statistical analysis of the correlation between the number of surgical operations and the results of tests on various aspects of intelligence.

| Test / Group | "Cattell IQ" | "Koos Cubes" | "Similarities" | "Vocabulary" | "10 Words" |
| --- | --- | --- | --- | --- | --- |
| **Full Sample (Spearman)** | p = 0.054 | p = 0.112 | p = 0.116 | p = 0.217 | p = 0.062 |
| **Elementary School Age (Spearman)** | p = 0.592 | p = 0.301 | p = 0.260 | **p = 0.021** rho = 0.656 | p = 0.034 |
| **Middle School Age (Spearman)** | **p = 0.045** rho = -0.453 | p = 0.685 | p = 0.165 | p = 0.886 | p = 0.037 |

*Note: Statistically significant p-values (p < 0.05) are highlighted in bold.*

Supplementary Table 14**.** Statistical analysis of differences in test results between children with preserved and impaired hand grasp function.

| Test / Group | "Cattell IQ" | "Koos Cubes" | "Similarities" | "Vocabulary" | "10 Words" |
| --- | --- | --- | --- | --- | --- |
| **Full Sample (Mann-Whitney U)** | p = 0.648 | p = 0.076 | p = 0.129 | **p = 0.044** | p = 0.580 |
| **Elementary School Age (Mann-Whitney U)** | p = 0.122 | **p = 0.027** | p = 0.080 | p = 0.156 | p = 0.060 |
| **High School Age (Mann-Whitney U)** | p = 0.121 | p = 0.495 | p = 0.109 | p = 0.086 | p = 0.580 |

*Note: Statistically significant p-values (p < 0.05) are highlighted in bold. The "Vocabulary" test result for the full sample showed higher scores in the group with *impaired* grasp.*
